# Sexting, Mental Health, and Victimization Among Adolescents: A Literature Review

**DOI:** 10.3390/ijerph16132364

**Published:** 2019-07-03

**Authors:** Aina M. Gassó, Bianca Klettke, José R. Agustina, Irene Montiel

**Affiliations:** 1 Faculty of Law, Universitat Internacional de Catalunya, 08017 Barcelona, Spain; 2 School of Psychology, Deakin University, Burwood, Victoria 3125, Australia

**Keywords:** adolescents, sexting, child victimization, mental health, threshold

## Abstract

The practice of creating and sharing sexual images via technological devices, known as sexting, has received crescent attention in the past years, especially due to the increase of adolescent engagement in this behavior. Although consensual sexting is not prima facie a crime, as some research has shown, it has the potential to be a risky behavior, and a threshold to get exposure to dangerous kinds of victimization as sextortion, online grooming or cyberbullying. In this context, teenagers represent a vulnerable group due to their limited ability of self-regulation, their high susceptibility to peer pressure, their technophilia, and their growing sexual curiosity. The present paper aims to review the scientific literature to analyze the relationship between mental health and sexting as a potentially risky behavior and its association with online victimization. The results and implications will be discussed.

## 1. Introduction

The term sexting was first used in 2005 by the Daily Telegraph, to unify the terms “sex” and “texting” and became an official word in 2009 [1]. It is generally known as “Sending and receiving sexual content (e.g., photos, videos) via the Internet and mobile phones” [2], but there is no consensus around the definition of the term sexting in the scientific community. Therefore, it has been diversely defined, including from broad definitions that include the sending of any kind of sexual content to narrower definitions, which are image-based only [3]. Some authors include coercion as part of the sexting behavior [4], while others consider that sexting is voluntary by definition [5]; some definitions include sending text messages (non-image based) as part of the sexting behaviors [6,7], while others exclude them from the definition [8,9]. The existing literature on sexting also differs in the population samples used for the research (teens vs. adults), and in the items used to measure sexting, which might be some of the reasons for the lack of a unified definition of the term. 

The research on sexting has widely grown over the past few years, especially regarding adolescents and the negative effect it might have on their sexual development and mental health, specifically after the publication of the Sex and Tech Survey (2008) results, which was the first broad survey to examine this phenomenon [10,11]. There is a conceptual debate in the scientific community that distinguishes between two clear arguing lines: one side tends to argue for a normalizing discourse whereby authors believe sexting to be a normative behavior as a part of sexual expression in a relationship [10,12], and it is possible to practice “safe sexting” to avoid negative consequences. The other side tends to argue that sexting is a risky behavior that requires intervention and prevention to diminish its prevalence, and has been labeled as “deviance discourse” [10,13,14,15]. Although sexting is a common behavior among the adolescent and young population, the deviance discourse seems to have more scientific support than the normalizing discourse. For example, a recent meta-analysis published by Kosenko et al. [16] found a significant relationship between sexting and three aspects of sexual behavior: general sexual activity, unprotected sex history, and number of sexual partners, that are all considered sexual risk behaviors. Similarly, a recent meta-analysis by Mori, Temple, Browne, and Madigan [17] has indicated that sexting behaviors were significantly associated with sexual behaviors, such as sexual activity, having more than one sexual partner, and lack of contraception use. It was also found that sexting behaviors and internalizing problems, such as anxiety and depression, were significantly associated. Importantly, the younger the adolescents, the stronger the observed association. 

Klettke et al. [18] in their literature review found significant relationships between sexting and risky sexual behavior and with several other adverse outcomes, such as (a) the sharing of sexual content without consent, (b) legal consequences, and (c) negative mental health repercussions [18]. Furthermore, research highlights an existing relationship between mental health or psychological health and online victimization behaviors, such as cyberbullying, online dating violence or revenge porn [18,19,20,21], which are closely related to sexting [5,15,22,23]. 

Several studies have identified a relationship between cyberbullying and sexting behaviors [24,25]. Fahy et al.’s [19] investigation emphasizes the high prevalence of cyberbullying and the potential of cyber-victimization as a risk factor for future depressive symptoms, social anxiety symptoms, and below average well-being among adolescents. Their results show that cyber-victims and cyberbully-victims were significantly more likely to report symptoms of depression and social anxiety. According to these results, it would be expected that sexting behaviors as a form of victimization might also be related to a higher likelihood of reporting depressive and anxiety symptoms. Along the same line of reasoning, research findings indicate that a higher degree of depressive symptoms is associated with greater Internet use [26,27], and a more frequent and problematic internet use is associated with higher rates of sexting behavior [27,28]. Therefore, it would be reasonable to hypothesize that higher engagement in sexting behaviors might predict higher rates of depressive symptoms. 

Considering the increasing number of suicides related to sexting [29], the relationship between sexting and mental health seems of particular interest, even though results up to date are somewhat mixed [18,29]. A few studies have investigated personality traits and their relationship with sexting [2,30]; others have explored the relationship between sexting and sexual risky behaviors or substance abuse and emotional problems [8,31,32,33]. However, only a few studies have investigated the relationship between negative mental health symptoms and sexting [4,34,35]. Discrepancies found in the literature may be due to differences in the definition of sexting, its measurement, methodologies or even due to the difference between those teens that sext consensually versus those who are pressured into sexting [29,36]. For the purpose of the present review, Wolak and Finkelhor’s conceptual framework of sexting will be used [37]. According to these authors’ typology, sexting behaviors can be divided into two broad categories: aggravated sexting and experimental sexting. Aggravated sexting behaviors encompass all types of sexting that may involve criminal or abusive elements beyond the creation, sending or possession of youth-produced sexual content, including (1) adult involvement; or (2) criminal or abusive behavior by minors. On the other hand, experimental sexting behaviors comprise those instances that do not include abuse or coercion, whereby teens voluntarily took pictures of themselves to create flirting or romantic interest in others. 

The main research questions this narrative review seeks to answer is: Is there a relationship between teen sexting behaviors and mental health? And, if so, which negative mental health impacts have been found when teens engage in sexting behaviors? Considering this, the present study aims to review research studies which have explored mental health variables associated with sexting behaviors and whether a significant relationship between sexting and negative mental health symptoms has been found. If sexting were found to have a negative mental health impact on adolescents, these results could have important implications to inform prevention campaigns targeted at schools, parents, educational communities, and healthcare providers.

## 2. Method 

In previous years, the body of research regarding sexting behaviors has increased dramatically, especially research focused on adolescent and teenage population. As such, several studies have highlighted that sexting behaviors increase as adolescents grow older [2,10]. Furthermore, an extended body of literature suggests that some sexting behaviors (e.g., sending or distributing) can be a risk behavior that can lead to or be seen as a form of online victimization of those depicted in the images, similarly, to cyberbullying or grooming [15,20]. On the other hand, the number of studies exploring the relationship between sexting and psychological variables has been growing in the past years, focusing especially on young adults or adult population [2,18,28], even though up to date there have been no conclusive results on the matter. For this reason, this narrative review aims to identify both empirical and non-empirical research addressing the relationship between sexting behaviors among teenagers and mental health. We consider this topic to be of considerable relevance to parents, the education community, and health care practitioners working with young people who engage in this behavior. 

Criteria for the inclusion in the review were as follows:

- Research (either empirical or non-empirical but excluding doctoral dissertations) exploring sexting behaviors amongst adolescent population between the ages of 10 and 21 years old.
- Examination of the relationship between sexting behaviors and mental health variables either as predictors or as consequences.
- Discussions around any psychological consequences related to young people’s sexting practices, emotional well-being or psychosocial health.

The following databases were searched: SCOPUS, PsycInfo, MEDLINE, and PUBMED, using the key words “sexting” AND “mental health”, “anxiety”, “depression”, and “psychology”. In addition, reference lists of reviewed articles were examined in relation to the topic of search, such as the one found in [29].

**Figure 1:**
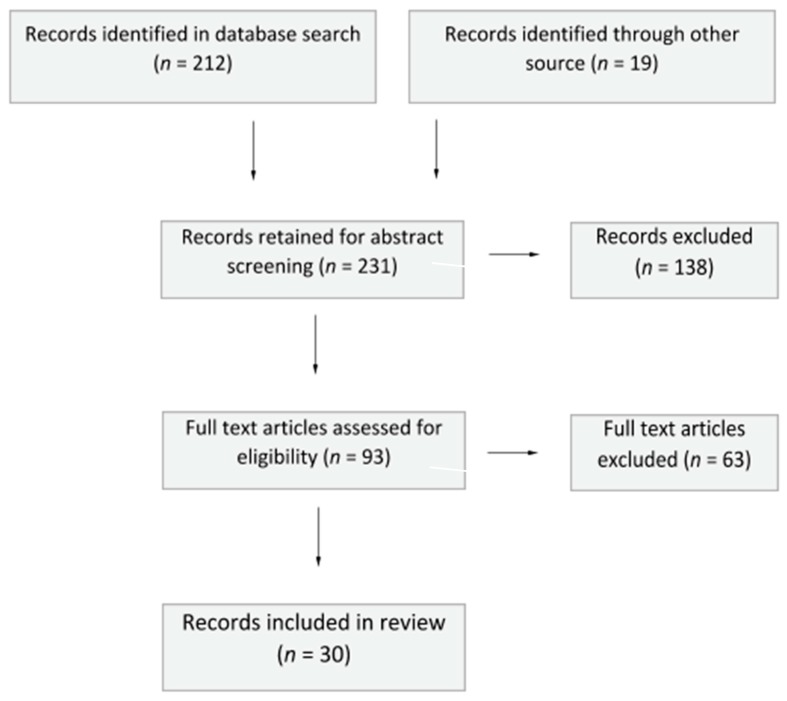
Flow chart of record identification, selection, and inclusion of articles.

Keeping in mind the extensive body of existing literature and the continually changing nature of online media technology-related research, the review was restricted to search literature published between January 2012 and March 2019, written either in English or Spanish, and appearing in peer-reviewed journals. The search was conducted in April 2019. A visual summary of the process is presented as a flow chart in Figure 1.

As exclusion criteria, those articles that did not include the review topics in their abstracts or were not directly related to the topic were excluded from the review. For instance, studies investigating sexting prevalence or mental health variables in adults, or mental health variables associated to other forms of victimization, such as bullying, were excluded. 

The initial bibliographic database search produced 212 articles. In addition to this, 19 articles were added following hand-searches through reference lists. These 231 articles were included in the first review and were screened by title and abstract. A total of 138 articles were excluded for not meeting the inclusion criteria, as they did not address the key areas of interest. 

The remaining 93 articles were then assessed for eligibility based on their full text. At this point, any articles that focused on mental health related to sexting in an adult population were excluded. Similarly, articles were excluded if they focused on information regarding new technologies, social media, sexting or cyberbullying in teenage population but did not relate to mental health. Finally, given the wide amount of international literature based on legal aspects of sexting, articles relating to this topic were excluded. This led to the exclusion of 63 more articles, for not meeting the relevant search areas. In total, 30 studies were identified for inclusion in this review. 

## 3. Results

### 3.1. Psychosocial Health and Sexting 

The results shown by Mitchell et al. [8] reveal that 21% of teens appearing or creating sexually explicit images and 25% of teens that had received such images reported feeling very or extremely upset, embarrassed or afraid as a result of their actions. Livingstone and Görzig’s [32] research focused on explaining the incidence of risk and harm reported by children and adolescents in relation to sexting behaviors. In a sample of 2036 European 11 to 16 year-olds reporting that they had received a sexual message on the Internet in the last 12 months, 24% responded “yes” when asked: “In the last 12 months, has any sexual message that you have seen or received bothered you in any way? For example, made you feel uncomfortable, upset, or feel that you should not have seen it?” [32]. Subjects who were younger, female, less sensation seeking, had pre-existing psychological difficulties and used the Internet less, were more likely to experience harm from the message. The details of the studies included in the review can be found in Table 1.

A study carried out by Ybarra & Mitchell [33] evaluating psychosocial problems from a sample of 3715 teens aged 13 to 18 years old, found that psychosocial problems were more frequently observed in teens who had sent or showed sexual photos of themselves. In addition, they found that high self-esteem was negatively associated with having sent or showed sexual pictures, and for female teens, results showed a significant association between sexting and depressive symptomatology. Similarly, Ševčíková [38] found that sexting was associated with emotional problems, and explored the possibility that this correlate might be both a predictor, as well as an outcome of sexting behaviors. 

Regarding sexting and personality, the research carried out by Gámez-Guadix et al. [2] shows an existing significant positive relationship between sexting and higher scores in Extraversion and Neuroticism and a negative relationship between sexting and Conscientiousness and Agreeableness. Brinkley et al. [30] conducted a study with a sample of 181 adolescents to evaluate the relationship between sexting and Borderline Personality Disorder (BPD), amongst other variables. Their results supported the hypothesis that sexting at age 16 would be associated with borderline personality feature at age 18. In addition, the authors affirm that their findings suggest that sexting may contribute to psychological distress for adolescents.

Following these results, many investigations have linked sexting behaviors to impulsivity and substance abuse problems. Döring [10] points out that sexting is related to impulsivity, bad judgment, sensation seeking, and problematic alcohol and drug use, as well as to suicide. This author considers sexting to be either a manifestation or moderator of problematic sexual behavior. On the other hand, Judge [39] defines sexting as an emotionally-driven behavior, that is often related to impulsivity and a lack of anticipation of adverse consequences. 

Englander [4], on the other hand, distinguished between pressured-sexters and non-pressured sexters, and her results show that pressured-sexters were more likely to report having problems during high school with excessive anxiety, although results were not statistically significant. Along the same line, Temple et al. [34], did not find sexting to be a marker of mental health. In their study, they evaluated 937 teens from Texas public high schools on rating scales for depression, anxiety, impulsivity, and a positive response for a history of substance use. Their results show that subjects who had sent naked pictures of themselves to someone else through text or email were more likely to score higher on scales of depression and impulsivity, as well as more likely to report a history of substance use. However, when the results were adjusted for prior sexual behavior, age, gender, race/ethnicity, and parent education, sexting was only related to impulsivity and high-risk behaviors, but not to depressive symptoms.

### 3.2. Sexting and Depression

When considering research regarding sexting and depression, specifically, the vast majority of studies have found a positive association between depressive symptoms and sexting behaviors. Out of a total of 14 publications addressing this issue, 12 found a positive association between sexting behaviors and depressive symptoms [4,6,24,29,31,34,35,36,38,41,42,43,45]. 

One such example is a study by Dake, Price, Maziarz & Ward [31] who conducted research based on 1289 middle school and high school students. Their results showed that being depressed, having contemplated or attempted suicide in the past year, or having been cyber or indirectly bullied were significantly correlated with sexting. Similarly, Van Ouytsel et al. [35] found a significant relation between teen sexting and depressive symptoms. These results are in line with those found by Chaudhary et al. [40], who conducted a study with 1760 teens and found that youth who reported sexting were significantly more likely to report symptomatology of depression and anxiety, as compared to those who did not report sexting. Specifically, their results showed that between 20% and 27% of youth who sexted had depressive symptoms. In addition, Bauman [39] in a book chapter regarding sexting and cyberbullying and mental health consequences, explains that young people involved in sexting had higher rates of suicidal thoughts than those who were not involved, and they also showed higher rates of high-risk behavior. 

Finally, Gámez-Guadix and De Santisteban [43] in a recent study carried out with 1208 Spanish adolescents between ages 12 and 16, found that a higher degree of depressive symptoms predicted a higher degree of sexting behaviors. At the same time, they found that teens who presented greater depressive symptoms were more likely to participate in sexting behaviors over time. Findings suggested a significant association between sexting behaviors and suicidal thoughts, suicide attempts, depressive symptoms, and feelings of sadness [31]. Teenage boys and girls who engaged in sexting behaviors showed a higher risk of reporting suicidal thoughts even after controlling for cyber victimization and depression [28]. One explanation for this relationship has been suggested by Medrano et al. [28], who proposed that the relationship between sexting behaviors and depressive symptoms may be partially mediated by cyber-victimization. The exchange of intimate photos or videos increases the risk of being victimized, not only by the direct sender of the image-based sexual content but by anyone who might have access to it, as teens might find themselves involuntarily exposed to unwanted sexual content [28].

However, some research has found no association between mental health symptoms and sexting behavior. Morelli and colleagues [6] conducted a study with 1334 teens and young adults between the ages of 13 and 30 years old, trying to assess the relationship between sexting, psychological distress, and online dating violence. Their results showed that a higher engagement in sexting was associated to a higher likelihood of offline and online dating violence. Moreover, their findings show no differences in psychological distress between people who sexted frequently and those who did not. Further, no relationship was found between sexting behaviors and symptoms of anxiety or depression. Finally, recent research conducted by Klettke and colleagues [45] based on 598 young Australian and Indian adults did not find an association between the sending of sexts, depression, or anxiety. However, higher levels of stress were significantly associated with the sending of sexts. Regarding gender, for males overall, higher levels of stress and lower levels of depression were associated with sending sexts, while for females, there were no associations with mental health variables. In terms of cultural differences, higher levels of stress were associated with sending sexts for participants overall, and for Indian respondents, but not Australians when analyzed separately.

One explanation for why some studies have not found an association may be due to the level of consent. Frankel et al. [29] collected data from a sample comprising 6021 US students between 9th and 12th grade to examine the relationship between consensual and non-consensual sexting and mental health. Their results show a correlation between consensual sexting and alcohol and tobacco use, being cyber-bullied and reporting both depressive symptoms and previous suicide attempts, especially in male respondents. Moreover, they found that non-consensual sexting was more prevalent among students who reported serious depressive symptoms, attempting suicide and self-harm. 

### 3.3. Sexting and Anxiety Symptoms 

Similar to the results observed regarding the analysis of the relationship between sexting behaviors and depression, the existing literature was reviewed to explore the relationship between sexting behaviors and symptoms of anxiety. Research exclusively investigating the relationship between these two variables is scarce; however, the majority of studies have found an existing relationship between the two variables. Out of a total of eight studies [4,6,24,36,40,41,42,45] seven studies found a positive association between sexting behaviors and symptoms of anxiety. 

For example, Chaudhary and colleagues [40] found that youth who reported having engaged in sexting behaviors, based on 1760 teens, were significantly more likely to report symptomatology of anxiety. Their results show that between 57% and 61% of adolescents who sexted had symptoms of anxiety. Similarly, Cooper et al. [41] reported that sexting victimization corresponded with negative psychological outcomes, including feelings of sadness, anger, and anxiety disorders. Finally, Klettke and colleagues [36] collected data from a sample comprising 444 late teens and found that receiving unwanted sexts and sending sexts under coercion was associated with poor mental health; they found that, especially, when receiving or sending unwanted but consensual sexts, respondents reported higher levels of depression, anxiety, stress, and lower self-esteem. 

## 4. Discussion

Research on sexting has grown widely over the past few years, especially regarding adolescents and the negative effect it might have on their sexual development and mental health, and specifically after the publication of the Sex and Tech Survey (2008) results [10,11]. Many studies have defined sexting as a form of victimization and have highlighted the potential for a relationship between victimization and mental health or psychological health and other online victimization behaviors, such as cyberbullying, online dating violence or revenge porn [18,19,20,21]. This review gathered the existing literature published from January 2012 to March 2019 that fit under the inclusion criteria (30 articles), to explore the relationship between sexting and mental health variables in the adolescent population. The relationship between these variables will be of interest to parents, educators, and the health care community to have a deeper understanding of the phenomena, so that appropriate prevention plans and campaigns, as well as intervention programs, can be developed and put into motion. 

Overall, and in line with Klettke et al.’s [18] results, the evidence regarding the relationship between teen sexting and mental health symptoms remains scarce, to some degree inconclusive and heterogeneous, and there is little empirical evidence on half-term and long-term consequences on adolescents. While the majority of studies have found significant associations between sexting and mental health symptoms, others did not find significant results [34]. One of the reasons for these equivocal findings may be because most studies do not differentiate between consensual (experimental) and non-consensual (aggravated) sexting. This may be a critical factor, as it has been shown that psychological outcomes vary when it comes to sexual coercion [51]. Another reason might be that different studies focus on measuring different psychological variables which could be interrelated. For instance, some studies focused on measuring personality traits [2,30], while others measured emotional problems [38], psychosocial problems, self-esteem, or depressive symptoms [33]. In addition, some studies explored the psychological variables as predictors of sexting [32,35], while others measured them as consequences of the behavior [8]. 

However, in general, findings suggest a significant association between sexting behaviors and suicidal thoughts, suicide attempts, depressive symptoms, and feelings of sadness [31]. Teenage boys and girls who engage in sexting behaviors have shown a higher risk of reporting suicidal thoughts even after controlling for cyber victimization and depression [28]. A possible explanation for this relationship is that both sexting behaviors and suicidal thoughts are risky behaviors in the adolescent population and tend to appear in conjunction [52]. 

The present review showed that 12 out of the 14 reviewed studies found a relationship between sexting behaviors and depressive symptoms, and findings lead to the conclusion that this relationship may be bi-directional. Gámez-Guadix and de Santisteban [43] argue that depressive symptoms and low self-esteem can predict sexting over time because sexting might be a way for teens to feel considered and desired. Moreover, they argued that adolescents with depressive symptoms might have fewer coping skills when pressured by peers to engage in sexting, which would explain why teenagers who sexts reported more depressive symptoms than those who do not. 

According to Medrano et al. [28], the relationship between sexting behaviors and depressive symptoms may be partially mediated by cyber-victimization. The exchange of intimate photos or videos increases the risk of being victimized, not only by the direct sender of the image-based sexual content but by anyone who might have access to it [28]. 

Similarly, seven out of the eight reviewed articles found a relationship between sexting behaviors and anxiety symptoms. Chaudhary et al. [40] found a significant relationship between youth who sexted and anxiety symptoms and argue that their results might be contrary to other findings due to the young age of the participants. This supports results obtained by Klettke et al. [36] who found a relationship between sexting and anxiety in an older sample but only related to receiving unwanted or sending unwanted but consensual sexts. 

## 5. Conclusions

The findings of this narrative review seem to point towards the presence of mental health symptomatology, particularly depression and anxiety, in the adolescent population when related to sexting behaviors. However, the age of the adolescents also seems to play an important role, as observed by Mori et al [17]. As adolescents get older, mental health symptoms seem to be increasingly associated with aggravated sexting, but not when related to consensual sexting behaviors whereby older teens have not been pressured (experimental sexting) [36,37,51]. Therefore, it might be probable that the relationship between sexting and poor mental health, depression, and anxiety symptoms is mediated by coercion, victimization, and age. Future research is needed to explore this hypothesis further. 

Despite the inconclusive results, this review shows that psychological aspects are related in some way to sexting, potentially as predictors of sexting behavior or as consequences, however, especially when taking into consideration sexting coercion or victimization. This finding is relevant because it can help raise awareness about the fact that sexting in adolescent populations should be further studied to establish effective mental health response programs and prevention programs, and that in some individual cases it might be a risky or dangerous behavior for teens to engage in or an indicator of some form of victimization. Along the same line, these findings suggest that both parents and educational communities should pay attention to both psychological symptoms and sexting behaviors since one can be a predictor of the other and vice versa. 

## Figures and Tables

**Table 1:** Details of studies included in the review.

Author	Type of Article	N (% Women)	Age Range	Definition of Sexting	Results (Mental Health)
Ahern and Mechling [38]	NE	-	-	“Sexting (texting plus sex) includes behaviors, such as sending, receiving, or forwarding of nude or partially nude images via cell phones”	Sexting may be associated with depression, contemplation of/attempted suicide, or a victimization of physical abuse or cyberbullying [31]
Bauman [39]	NE	-	-	“The term sexting refers to the practice of transmitting sexual content via digital technology and includes images, video, and text”. According to this author, sexting is a form of cyberbullying when “the messages or images are used to inflict harm on a target by causing humiliation and embarrassment”	Targets of cyberbullying and young people involved in sexting had higher rates of suicidal thoughts than those who were not involved, and they also had higher rates of high-risk behaviors (alcohol, drugs, stealing).
Brenick, Flannery, and Rankin [15]	E	169 (80)	18–25	“The sending or receiving of sexually suggestive written messages, pictures, or videos”	The authors found three significant predictors of sexting experiences and evaluations of sexting and sexting victimization: anxious and avoidant attachment and rejection sensitivity.
Brinkley, Ackerman, Ehrenreich, and Underwood [30]	E	181 (46.9)	15–16	“Sexting refers to sending sexually explicit or suggestive images, videos, or text messages via digital communication”	Sending sex texts at age 16 predicted for borderline personality traits at age 18.
Chaudhary, Peskin, Temple, Addy, Baumler, and Shegog [40]	E	1760 (52.4)	M = 12.2	“The practice of sending or posting sexually suggestive text messages, videos, and images, including nude or semi-nude photographs or videos, via cellular telephones or over the Internet (such as email or social networking sites, such as Facebook)”	Authors found that youth who reported sexting were significantly more likely to report symptomatology for depression and anxiety as compared to those who did not report sexting (between 20% and 27% of youth who sexted had depression, and between 57% and 61% of youth who sexted had anxiety.
Cooper, Quayle, Jonsson, and Svedin, [41]	NE	-	-	“Sending or posting of sexually suggestive text messages and images, including nude or semi-nude photographs, via mobiles or over the Internet”	Research findings suggest a link between sexting behaviors and higher rates of problematic alcohol and recreational drug use. Dake et al. [31] found a correlation between self-producing and sending sexual images and being depressed, having contemplated or attempted suicide in the past year, having been cyber or indirectly bullied, and having encountered physical force within a relationship. Victimization corresponded with negative psychological outcomes including feelings of sadness, anger and anxiety disorders as well as depression and ultimately, suicide.
Dake, Price, Maziarz, and Ward, [31]	E	1289 (48)	12–18	“Sending, receiving, or forwarding sexually explicit messages or nude, partially nude, or sexually suggestive digital images of one’s self or others via a cell phone, e-mail, Internet, or SMS”	Associated with sexting: emotional health issues, including being depressed, having contemplated or attempted suicide in the past year, having been cyber or indirectly bullied, and having encountered physical force used against the student in the form of being hit by a boyfriend or girlfriend or being forced to have sexual intercourse.
Döring [10]	NE	-	-	“The private exchange of self-produced sexual images via cell phone or the internet”	Sexting is related to suicide. Sexting behavior is placed in a context of adolescent impulsivity, bad judgment, sensation seeking, and problematic alcohol and drug use. Sexting is seen as a manifestation or moderator of problematic and age-inappropriate sexual behavior.
Englander, E. [4]	E	617 (-)	18 only	“Sending nude pictures of yourself”	Sexters had less depression than non-sexters but more anxiety. Relationship not significant.
Eugene, [42]	NE	-	-	“Sending or showing someone sexual pictures of yourself nude or nearly nude”	Sexting linked to risky sexual behaviors and a number of psychosocial issues, such as depression, anxiety, and low self-esteem.
Frankel, Bass, Patterson, Dai, and Brown, [29]	E	6021 (49.4)	14–18	“To share nude, sexually explicit, or sexually suggestive photos via text or social media platforms”	Significant relationship found between consensual sexting and depressive symptoms, suicide attempt and self-harm, but depressive symptoms were more prevalent in students who reported non-consensual sexting.
Gámez-Guadix and De Santisteban. [43]	E	1208 (52.8)	12–16	“The voluntary creation and delivery of text messages, photos, or videos, with personal sexual content via the Internet or mobile devices”	The authors found that more depressive symptoms predicted more sexting. Regarding psychological adjustment, adolescents presenting more depression symptoms tended to participate more in sexting over time.
Gámez-Guadix, de Santisteban, and Resett, [2]	E	3223 (49.9)	12–17	“The voluntary creation and delivery of text messages, photos, or videos, with personal sexual content via the Internet or mobile devices.”	The personality profile of those involved in sexting was characterized by higher Extraversion and Neuroticism and by lower scores in Conscientiousness and Agreeableness.
Holoyda, Landess, Sorrentino, and Friedman, [27]	NE	-	-	“It generally involves the transmission of text, pictures, or videos containing sexual material”	The evidence regarding the relationship between teen sexting and specific psychiatric disorders or psychological sequelae remains scant and inconclusive.
Judge, [44]	NE			“The exchange of sexually explicit images between adolescents via cell phone”	Sexting may be viewed as an emotionally driven behavior that is often impulsive and without a clear anticipation or understanding of the potential adverse consequences.
Klettke, Hallford, and Mellor, [18]	NE	-	-	-	While some findings indicate sexting behavior as being associated with lower well-being or higher psychological distress, findings across the literature appear to be mixed.
Klettke, Hallford, Clancy, Mellor, and Toumbourou, [36]	E	444 (50.7)	18–21	“The sending, receiving, or forwarding of sexually explicit messages, images, or photos to others through electronic means, primarily between cellular phones”	The results showed that having sent or received sexts was not associated with any psychological variables. Receiving unwanted sexts and sending sexts under coercion were associated with poorer mental health. Specifically, when receiving or sending unwanted but consensual sexts, respondents reported higher depression, anxiety, and stress, and lower self-esteem.
Klettke, Mellor, Silva-Myles, Clancy and Sharma, [45]	E	598 (75.5/56.3)	17–21	“Sending, receiving or forwarding of sexually explicit messages, images or photos to others through electronic means, primarily between cellular phones”	Only higher levels of stress were significantly associated with sending sexts, not depression or anxiety.
Korenis and Billick, [24]	NE	-	-	“Sexting refers to the practice of sending sexually explicit material including language or images to another person’s cell phone”	Depression, suicide, mood disorder, adjustment reactions, and anxiety disorders are some of the potential psychiatric sequelae of falling victim to sexting.
Livingstone and Görzig, [32]	E	18,709 (50)	11–16	“The peer-to-peer exchange of sexual messages using digital technologies (known popularly as sexting). Such messages may be created and exchanged via text or image messaging on mobile phones, though they also include peer-to-peer messaging on diverse internet-enabled devices, particularly using social networking sites and instant messaging services.”	The risk of receiving sexually explicit images was higher for those with psychological difficulties. Adding the behavioral variables reduced the effect of the psychological variables and age, suggesting that the behavioral variables mediate the effect of the psychological variables and age.
Lorang, McNiel, and Binder, [46]	NE	-	-	“Sexting is the sending or forwarding of sexually explicit photographs or videos of the sender or someone known to the sender via cell phone”	Sexting cases followed by suicide.
Mitchell, Finkelhor, Jones, and Wolak, [8]	E	1560 (50)	10–17	“Sexting generally refers to sending sexual images and sometimes sexual texts via cell phone and other electronic devices”	21% of respondents appearing in or creating images reported feeling very or extremely upset, embarrassed, or afraid as a result of engaging in sexting, as did 25% of youth receiving images.
Morelli, Bianchi, Baiocco, Pezzuti, and Chirumbolo, [6]	E	1334 (68)	13–30	“Sexting is the exchange of sexually explicit or provocative content (text messages, photos, and videos) via smartphone, Internet, or social networks.”	Results showed that high/moderate users of sexting committed more offline and online dating violence. Regarding psychological distress, no differences were found between high and low/moderate users of sexting. No relationship with anxiety and depression symptoms.
Moreno-Bernal, Valdez-Montero, Gámez-Medina, and Cortez, [47]	NE	-	-	“The act of sending, receiving or publishing sexually provocative or explicit messages, images or videos through a mobile phone or social media”	Studies showed that the practice of sexting is increased by consuming some type of drug, as well as engaging in risky sexual behaviors.
Ševčíková, [48]	E	17,016 (50)	11–16	“Sexting refers to the electronic exchange of sexually suggestive messages (i.e., sexts), mainly pictures depicting their authors in nude or semi-nude positions”	Having more emotional problems was associated to having a higher likelihood of involvement in sexting behavior. Sexting might not necessarily be a marker of poor mental health.
Smith, Thompson, and Davidson, [49]	NE	-	-	“The sending, receiving, and forwarding of sexually explicit messages, images or photos to others through electronic means, primarily between cellular phones’	Predictors of risk of harm from receiving sexts are being younger, female, and scoring higher on psychological difficulties and lower on sensation seeking. Other predictors of involvement in sexting are being sexually active, involvement in alcohol and drug use, having unprotected sex, engaging in web-based chatting with strangers and viewing adult pornography and personality variables of neuroticism and low agreeableness.
Temple, Le, van den Berg, Ling, Paul, and Temple, [34]	E	937 (57)	14–18	“Electronically sending sexually explicit images from one adolescent to another”	Significant association between sexting and symptoms of depression, impulsivity, and substance abuse but not when adjusted for other variables: sexting is not a marker of mental health.
Van Ouytsel, Van Gool, Ponnet, and Walrave, [35]	E	1028 (58)	15–18	“Sending sexually explicit pictures through the internet or the mobile phone”	Significant relationship between depression and engagement in sexting.
Van Ouytsel, Walrave, Ponnet, and Heirman, [50]	NE	-	-	“The exchange of sexually explicit content communicated via text messages, smartphones, or visual and web 2.0. activities, such as social networking sites”	Adolescents who engaged in sexting were more likely to ever have become victims of traditional forms of bullying [31]. Youth who engaged in sexting had lower awareness and understanding of their emotions and experienced more difficulties with regulating their emotions. And an association between sexting and impulsivity was found.
Ybarra and Mitchell, [33]	E	3715 (56.6)	13–18	“Sending and sharing sexual photos online, via text messaging, and in person”	Adolescents who sexted were more likely to use substances and less likely to have a high self-esteem.

Note: E = empirical study, NE = non-empirical study; - = no data found
